# The study of *t*-*PA*, *u*-*PA* and *PAI*-*1* genes polymorphisms in patients with abdominal aortic aneurysm

**DOI:** 10.1007/s11033-014-3141-6

**Published:** 2014-01-23

**Authors:** Katarzyna Oszajca, Konrad Wroński, Grażyna Janiszewska, Małgorzata Bieńkiewicz, Jacek Bartkowiak, Janusz Szemraj

**Affiliations:** 1 Department of Medical Biochemistry, Medical University of Lodz, Mazowiecka 6/8 Street, 92-215 Lodz, Poland; 2 General and Vascular Surgery Department, M. Pirogow Regional Specialist Hospital, Wolczanska 195 Street, 90-531 Lodz, Poland; 3 Department of Quality Control and Radiological Protection, Medical University of Lodz, Czechoslowacka 8/10 Street, 92-216 Lodz, Poland

**Keywords:** Abdominal aortic aneurysm, *t*-*PA* −7351 C/T polymorphism, *u*-*PA* 1788 C/T polymorphism, *PAI*-*1* −844 G/A polymorphism, *PAI*-*1* −675 4G/5G polymorphism, RFLP

## Abstract

The most important feature of abdominal aortic aneurysm (AAA) pathogenesis is an enzymatic degradation of elastic lamellae and extracellular matrix proteins particularly with participation of matrix metalloproteinases. Plasmin, which is responsible for the dissolution of fibrin in blood vessels, plays also a key role in the cascade for activation of the metalloproteinases. The purpose of this study was to evaluate the influence of selected polymorphisms in genes coding for tissue plasminogen activator (−7351 C/T polymorphism), urokinase-type plasminogen activator (1788 C/T polymorphism) and plasminogen activator inhibitor 1 (−675 4G/5G and −844 G/A polymorphism) on the susceptibility to AAA. We performed a case–control study of 153 polish patients hospitalized due to AAA and compared them with matched healthy control subjects. The polymorphisms were ascertained through genotyping by polymerase chain reaction and restriction digestion of amplified fragments or through high-resolution melting analysis. In this study we have found lower frequency of wild-type GG genotype of the −844G/A *PAI*-*1* polymorphism in cases than in controls, what may suggest the protective effect of this genotype for the risk of AAA development. None of the remaining polymorphisms tested were associated with AAA occurrence.

## Introduction

Fibrinolytic system is a proteolytic cascade of activation events responsible for the enzymatic dissolution of fibrin blood clots. It is composed of plasminogen, its active form—plasmin, plasminogen activators—tissue-type (t-PA) and urokinase type (u-PA), inhibitors which regulate activation of plasminogen—plasminogen activator inhibitor type 1(PAI-1) and type 2 (PAI-2) and activity of plasmin—α2-antiplasmin. Plasmin, which is formed from plasminogen by t-PA or u-PA, is an enzyme responsible for the dissolution of fibrin in blood vessels, but also plays a key role in the cascade for activation of matrix metalloproteinases (MMPs) [1, 2]. MMPs could play an essential role in the destruction of extracellular matrix (ECM) in the aortic wall. Whereas an enzymatic degradation of elastic lamellae and ECM proteins, particularly with participation of MMPs, is the most important feature of abdominal aortic aneurysm (AAA) pathogenesis. AAA represents a degenerative life-threatening process of the abdominal aorta. Previous studies have found elevated levels of plasmin, as well as its activators in AAA tissue compared with controls [3–7]. It is also known that overexpression of *PAI*-*1* may prevent the development of AAA [8].

Tissue type plasminogen activator is mainly involved in intravascular thrombolysis, while urokinase mediates pericellular proteolysis during cell migration, wound healing, and tissue remodeling under physiological and pathological conditions [9–11]. −7351 C/T single nucleotide polymorphism of the gene encoding t-PA (rs63020761) is located within the enhancer region and the substitution of cytosine for thymine at position −7351 reduces the Sp1/Sp3 binding affinity resulting in the inhibition of DNA transcription [12]. This polymorphism was found to be strongly correlated with reduced endothelial t-PA release [13] and associated with the occurrence of myocardial infarction and stroke [14, 15]. A genetic polymorphism of *u*-*PA* consisting in a transition of cytosine to thymine at position 1788 (rs2227564) causes a missense mutation (Pro141Leu) in the kringle domain of uPA protein, which has been shown to decrease the affinity for fibrin [16]. This polymorphism has been associated with asthma, Alzheimer’s disease, colorectal cancer and *Helicobacter pylori* infection [17–21].

PAI-1 belongs to the serpin superfamily of proteins and is the main regulatory protein of the fibrinolytic system. In addition to its role as a regulator of haemostasis, PAI-1 also plays a significant role in several biological processes which include: wound healing, atherosclerosis, such metabolic disturbances as obesity and insulin resistance, tumor angiogenesis, chronic stress, bone remodeling, asthma, rheumatoid arthritis, fibrosis, glomerulonephritis, sepsis, and others [22, 23]. Both −675 4G/5G insertion/deletion and −844 G/A promoter polymorphisms of *PAI*-*1* gene cause the elevated PAI-1 level [24, 25]. The deletion of one guanine nucleotide at position −675 in the *PAI*-*1* promoter results in the disappearance of one binding site for a DNA-binding protein that acts as a transcriptional repressor [26, 27]. Thus, 4G allele has higher transcriptional activity than 5G allele. Whereas, an exchange from G to A at position −844 in the *PAI*-*1* gene promoter includes a consensus sequence binding site for the Ets nuclear protein and can be implicated in the regulation of the *PAI*-*1* gene expression [28, 29].

The purpose of our study was to examine the occurrence of selected polymorphisms in genes coding for t-PA (−7351 C/T), u-PA (1788 C/T) and PAI-1 (−675 4G/5G insertion/deletion and −844 G/A) in patients suffering from AAA.

## Materials and methods

### Patients

The study population was comprised of 153 Polish patients with AAA, who were hospitalized in the M. Pirogow Regional Specialist Hospital in Lodz. Control group consisted of 152 healthy volunteers who had no history or symptoms of aneurysms and other cardiovascular diseases. The study was approved by Ethics Committee of Medical University of Lodz (consent number: RNN/19/07/KE). All subjects gave written informed consent to participate.

### DNA isolation and genotyping

Venous blood samples were taken from all study participants. Genomic DNA was isolated from leukocytes obtained from whole blood samples using phenol–chloroform extraction method or QIAamp DNA Blood Mini Kit (Qiagen, Hilden, Germany).

*u*-*PA* 1788 C/T, *PAI*-*1* -844 G/A and *PAI*-*1* -675 4G/5G polymorphisms were genotyped using polymerase chain reaction-restriction fragment length polymorphism sites (PCR–RFLP). *t*-*PA* −7351 C/T polymorphism was determined by melting curve analysis using Light Scanner™ HR I 96 (Idaho Technology, Inc.). The PCR primer pairs, annealing temperature, PCR product sizes and restriction enzymes are listed in Table 1.

**Table 1:** Details of the PCR–RFLP conditions for SNP analysis

Polymorphism	Primers sequence	Annealing temp. (°C)	Product size (bp)	Restriction enzyme
*u*-*PA* 1788 C/T	5′ CTATCTTTCTGTGTTGCCAG 3′	58	197	*Alu*I
	5′ CCTCAGTCCTCCTGTGATGG 3′			
*PAI*-*1* −844 G/A	5′ CAGGCTCCCACTGATTCTAC 3′	60	510	*Xho*I
	5′ GAGGGCTCTCTTGTGTCAAC 3′			
*PAI*-*1* −675 4G/5G	5′ CACAGAGAGAGTCTGGCCACGT 3′	62	98/99	*Bsl*I
	5′ CCAACAGAGGACTCTTGGTCT 3′			
*t*-*PA* −7351 C/T	5′ TGAGTGATCTCATTGCCGAGG 3′	63 → 56	247	–
	5′ AGGTTGCAGTGAACTGTGATCC 3′			

PCR reactions for amplification of polymorphic segments were performed in reaction mixture containing 2 μl of 10× PCR buffer (with 15 mM MgCl_2_), 2 μl of 2.5 mM dNTPs, 100 pmol of each primer, 1 U of Taq DNA polymerase (Fermentas, Qiagen), 2 μl of genomic DNA and deionized water added to a final volume of 20 μl. Moreover, in the case of *PAI*-*1* −844 G/A polymorphisms there were additional 4 μl of 25 mM MgCl_2_ used. Basically, the amplification reactions were carried out as follows: 94 °C for 5 min, followed by 35 cycles of 95 °C for 30 s for DNA denaturation, 56–63 °C (depending on the DNA fragment, see the Table 1) for 30 s for primers annealing, 72 °C for 30 s for DNA extension and 72 °C for 10 min for final extension. Besides, the PCR cycles for amplification of *t*-*PA* −7351 C/T polymorphic site were run in different way: 95 °C for 30, 45 s for primers annealing at temperature lowering by 0.5 °C per each cycle from 63 to 56 °C (14 cycles and the remaining at 56 °C), 72 °C for 1 min. Amplification and correct sizes of PCR products were confirmed on polyacrylamide gels in Tris/acetate/EDTA buffer in comparison with 100 bp or 1 kb DNA Ladder (Fermentas). After staining with ethidium bromide the gels were photographed under UV light using Image Master VDS system (Pharmacia Biotech).

10 μl of the appropriate PCR product (for *u*-*PA* and *PAI* polymorphisms) was then digested overnight with an excess of proper restriction enzyme (see Table 1) under conditions recommended by the supplier (Fermentas). The digestion products were separated by electrophoresis on polyacrylamide gels, stained and visualized as described above. Molecular sizes of the restriction fragments are shown in Table 2.

**Table 2:** Molecular sizes of the restriction fragments

Polymorphism	Molecular sizes of restriction fragments (bp)		
	Wild-type homozygote	Heterozygote	Mutant homozygote
*u*-*PA* 1788 C/T	197	197, 124, 73	124, 73
*PAI*-*1* −844 G/A	364, 146	510, 364, 146	510
*PAI*-*1* −675 4G/5G	77, 22	98, 77, 22	98

In the case of *t*-*PA* −7351 C/T polymorphism, the 247 bp PCR product was then diluted 1:50 and used directly as DNA template for nested PCR with following primers: forward 5′ TAGGGCTTTGGCCGCTCTCCC 3′ and reverse 5′ GAGTCCCAGGCCATGGCTGTG 3′. The PCR reaction was performed in a DNA thermal cycler (Biometra) in 96-well plate coated with 20 μl of mineral oil. Each reaction mixture contained 4 μl of Light Scanner Master Mix (Idaho Technology, Inc.), 4 μl of water, 0.5 μl of each primer (100 pmol/μl) and 1 μl of DNA template. The conditions of the PCR were as follows: 94 °C for 5 min, followed by 20 cycles of 94 °C for 30 s for DNA denaturation, 60 °C for 30 s for primers annealing and 72 °C for 5 s for DNA extension. Amplified products of 60 base pairs were subjected to high-resolution melting analysis (HRM) in the same plate using the light scanner instrument.

To confirm the results obtained using PCR–RFLP and HRM technique, selected samples (every tenth) were subjected to DNA sequence analysis carried out by Genomed S.A. (Warsaw, Poland).

### Statistical analysis

The results are reported as percentages (%) or means with standard deviations (±SD). Analysis of association between genotype/allele distribution and AAA risk was performed with the use of the χ^2^ square test and the 95 % confidence intervals (CI) for disease odds ratio (OR) calculated with the use of logistic regression. All the analyses were derived by means of Statistica v8.0 software. The level of significance was set at *p* < 0.05.

## Results

The study group consisted of 51 females (33.3 %) and 102 males (66.6 %) with an age range from 44 to 73 years. The mean age in the study population was 59 years, SD = 7. The control group consisted of 152 healthy volunteers: 60 females (39.5 %) and 92 males (60.5 %) with their ages ranging from 37 to 70 (mean age 55, SD ±9). The control subjects had no history or symptoms of aneurysms and other cardiovascular diseases. The cases and controls were homogenous in gender and age distribution (*p* > 0.20).

We did not observe any statistical differences in the distribution of genotypes and alleles of *t*-*PA* −7351 C/T, *u*-*PA* 1788 C/T and *PAI*-*1* −675 4G/5G polymorphisms between AAA patients and healthy controls (*p* = 0.499, *p* = 0.628, *p* = 0.836, respectively) (Tables 3, 4, 5). Regarding the −844 G/A polymorphism of the *PAI*-*1* gene, we found significant differences in genotype frequency between cases and controls (χ^2^ = 7.88; df = 2; *p* = 0.020) (Table 6). The following genotype frequencies of the −844 G/A polymorphism were obtained in the study group: GG = 9.2 %, AG = 57.9 %, AA = 32.9 %, while these frequencies in the control group were 20.4, 47.6 and 32.0 %, respectively. However, the difference concerns only the frequency of GG genotype in AAA patients vs. controls (OR 0.40; 95 % CI 0.20–0.78) and the result achieved statistical significance (*p* = 0.008). Thus, it could be suggested that the GG wild-type genotype may have protective effect for the risk of AAA development.

**Table 3:** Distribution of frequencies of genotypes and alleles of *t*-*PA* −7351 C/T polymorphism in AAA patients and controls

	Cases (*n* = 153) *n* (%)	Controls (*n* = 150) *n* (%)	OR (95 % CI)	*p* value
Genotype				
CC	56 (36.6)	60 (40.0)	0.90 (0.54; 1.39)	0.54
CT	83 (54.3)	72 (48.0)	1.28 (0.82; 2.04)	0.28
TT	14 (9.1)	18 (12.0)	0.74 (0.35; 1.54)	0.42
Allele				
C	195 (63.7)	192 (64.0)	1.01 (0.67; 1.53)	0.95
T	111 (36.3)	108 (36.0)	0.99 (0.65; 1.49)	

χ^2^ = 1.39, df = 2, *p* = 0.499

**Table 4:** Distribution of frequencies of genotypes and alleles of *u*–*PA* 1788 C/T polymorphism in AAA patients and controls

	Cases (*n* = 152) *n* (%)	Controls (*n* = 152) *n* (%)	OR (95 % CI)	*p* value
Genotype				
CC	92 (60.5)	85 (55.9)	1.21 (0.76; 1.92)	0.42
CT	55 (36.2)	63 (41.5)	0.80 (0.50; 1.28)	0.35
TT	5 (3.3)	4 (2.6)	1.27 (0.33; 4.76)	0.73
Allele				
C	239 (78.6)	233 (76.6)	1.24 (0.76; 1.64)	0.56
T	65 (21.4)	71 (23.4)	0.89 (0.61; 1.31)	

χ^2^ = 0.93, df = 2, *p* = 0.628

**Table 5:** Distribution of frequencies of genotypes and alleles of *PAI*-*1* −675 4G/5G polymorphism in AAA patients and controls

	Cases (*n* = 145) *n* (%)	Controls (*n* = 152) *n* (%)	OR (95 % CI)	*p* value
Genotype				
4G/4G	41 (28.3)	39 (25.7)	1.14 (0.68; 1.92)	0.61
4G/5G	81 (55.9)	90 (59.2)	0.87 (0.55; 1.39)	0.56
5G/5G	23 (15.9)	23 (15.1)	1.05 (0.56; 2.01)	0.86
Allele				
4G	163 (56.2)	168 (55.3)	1.04 (0.75; 1.45)	0.82
5G	127 (43.8)	136 (44.7)	0.96 (0.69; 1.34)	

χ^2^ = 0.36; df = 2; *p* = 0.836

**Table 6:** Distribution of frequencies of genotypes and alleles of *PAI*-*1* −844 G/A polymorphism in AAA patients and controls

	Cases (*n* = 152) *n* (%)	Controls (*n* = 147) *n* (%)	OR (95 % CI)	*p* value
Genotype				
GG	14 (9.2)	30 (20.4)	0.40 (0.20; 0.78)	0.008
GA	88 (57.9)	70 (47.6)	1.52 (0.95; 2.38)	0.08
AA	50 (32.9)	47 (32.0)	1.04 (0.64; 1.69)	0.87
Allele				
G	116 (38.2)	130 (44.2)	0.78 (0.93; 1.78)	0.13
A	188 (61.8)	164 (55.8)	1.28 (0.93; 1.78)	

χ^2^ = 7.88, df = 2, *p* = 0.020

## Discussion

The pathogenesis of AAA has not yet been completely defined. The literature data show that proteolytic activity of fibrinolytic system may be one of the factors contributing to the development of aneurysms [4, 7]. Thus, the aim of the present study was an attempt of finding correlation between genetic polymorphisms of selected fibrinolysis genes and occurrence of AAA.

The TT genotype of −7351 C/T tissue-type plasminogen activator gene polymorphism has been reported to be associated with reduced t-PA release rates due to the decreased transcriptional activity [12, 13]. It has been documented that −7351 T allele confers an increased risk of such cardiovascular diseases as myocardial infarction [14] and lacunar stroke [15]. However, there are conflicting data concerning the contribution of −7351 C/T polymorphism and ischemic stroke. Tuttolomondo et al. [30] demonstrated a higher frequency of the CT genotype in cases but other researchers did not find any significant association of *t*-*PA* −7351C/T polymorphism with ischemic stroke [31, 32]. To our knowledge, there are no existing data concerning the occurrence of this polymorphism in individuals suffering from aneurysm. In this study we observed slightly higher frequency of CT genotypes in AAA individuals compared to controls but the difference did not achieve statistical significance (54.3 vs. 48.0 %, *p* = 0.28).

We have also studied, for the first time, the association between the 1788 C/T urokinase-type plasminogen activator gene polymorphism and risk of AAA. Since it is documented that elevated urokinase expression may participate in AAA formation and rapture [33–35], and that an exchange of cytosine to thymine at nucleotide 1788 decreases the urokinase activity, we expected to find a protective effect of the polymorphic T allele on the occurrence of the disease. But we did not find any significant correlation, although the frequency of T allele was slightly higher in controls than in cases (23.4 vs. 21.4 %).

Another fibrinolytic gene which we have studied regarding to its contribution to the aneurysm disease is plasminogen activator inhibitor 1. We have chosen two polymorphisms in the *PAI*-*1* gene promoter (−675 4G/5G and −844 G/A), that increase the *PAI*-*1* expression and plasma concentration. Elevated levels of PAI-1 decrease plasmin formation resulting in the inhibition of the proteolytic activity of MMPs, and thus could prevent the formation of aneurysm. Our study indicates a lower frequency of wild-type GG genotype of the −844 G/A *PAI*-*1* polymorphism in cases than in controls, what may suggest the protective effect of this genotype for the risk of AAA development. Although the −844 G/A *PAI*-*1* polymorphism influences the plasma PAI-1 level, there is at present a lack of studies regarding the association of this polymorphism and risk of AAA. However, there are some literature data on the relationship between this polymorphism and other vascular system diseases involving myocardial infarction [36], ischemic stroke [37] or deep vein thrombosis [28]. But all these studies have found no significant effect of individual genotypes on the risk of respective disease.

Our study revealed also the lack of correlation between −675 4G/5G *PAI*-*1* promoter polymorphism and AAA occurrence in Polish population. Nevertheless, there are some data concerning the correlation of 4G/5G *PAI*-*1* polymorphism with AAA. Jones et al. [38] noticed that this promoter polymorphism does not influence the development of AAA, although they observed faster AAA growth in patients with 5G/5G genotype. Similarly, Eriksson et al. [39] documented that patients with *PAI*-*1* 5G/5G genotype presented with AAAs of largest diameter. Whereas another report found the influence of 5G/5G genotype on the aneurysm development, but only in the case of typical familial AAA [40]. −675 4G/5G *PAI*-*1* promoter polymorphism is also reported to be associated with other cardiovascular diseases, including thrombosis [41], myocardial infarction [27, 36, 42] or coronary artery disease [43, 44].

In conclusions, in this study no association was observed between −7351 C/T *t*-*PA*, 1788 C/T *u*-*PA* and −675 4G/5G *PAI*-*1* polymorphisms and the risk of AAA development. Only in the case of −844 G/A *PAI*-*1* polymorphism the study revealed a slight difference in the wild-type genotype frequency between AAA patients and healthy controls. This result should be confirmed in a larger population.
